# Supplementary material for: Residual Cystine Transport Activity for Specific Infantile and Juvenile CTNS Mutations in a PTEC-Based Addback Model
Source: Cells. 2024 Apr 6;13(7):646. doi: 10.3390/cells13070646 (PMC11011962; doi:10.3390/cells13070646)
Supplement: Supplementary file 1 [file cells-13-00646-s001.zip › full-unedited-blots_V3.pptx]

## Slide 1
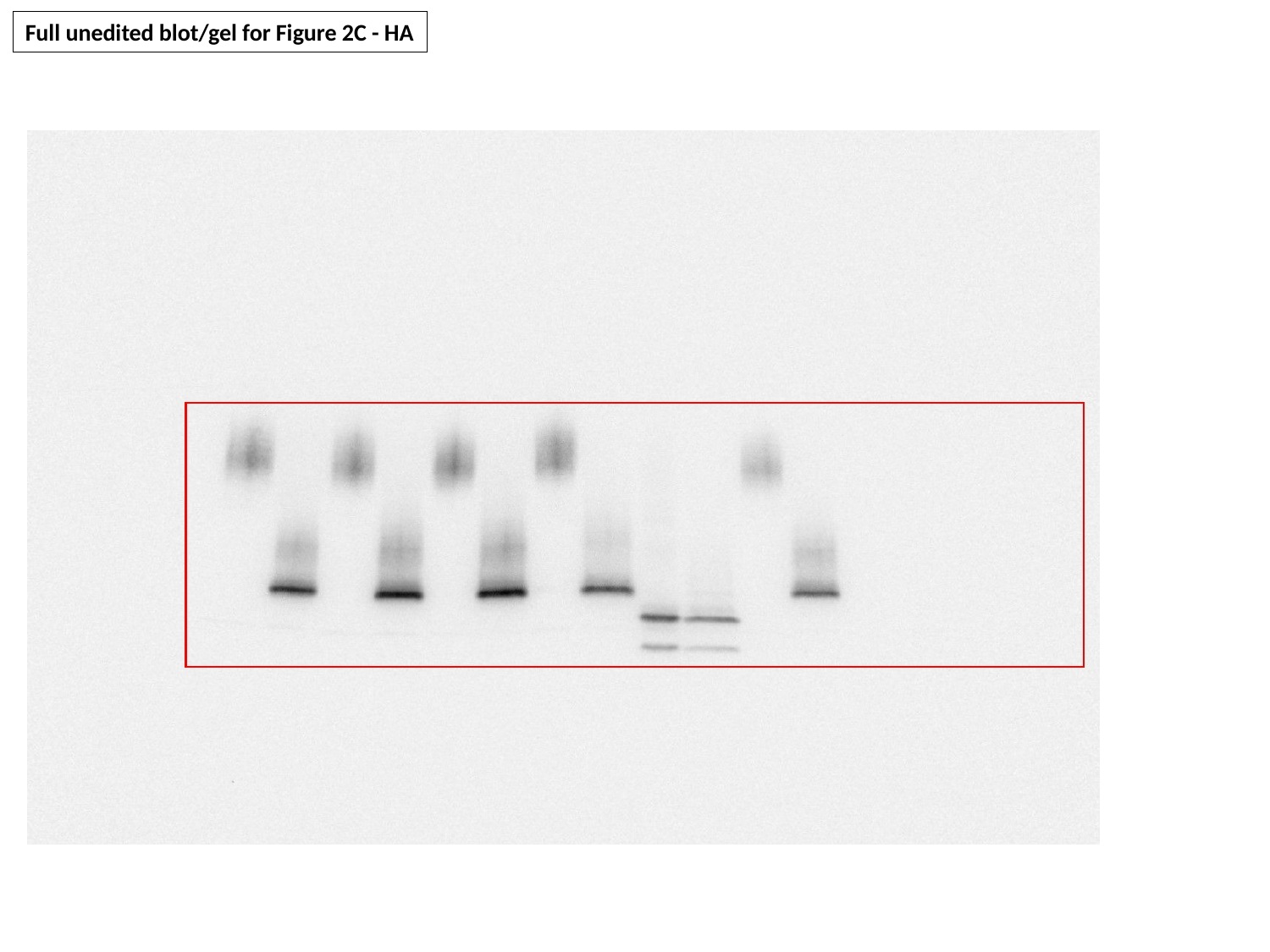

Full unedited blot/gel for Figure 2C - HA

## Slide 2
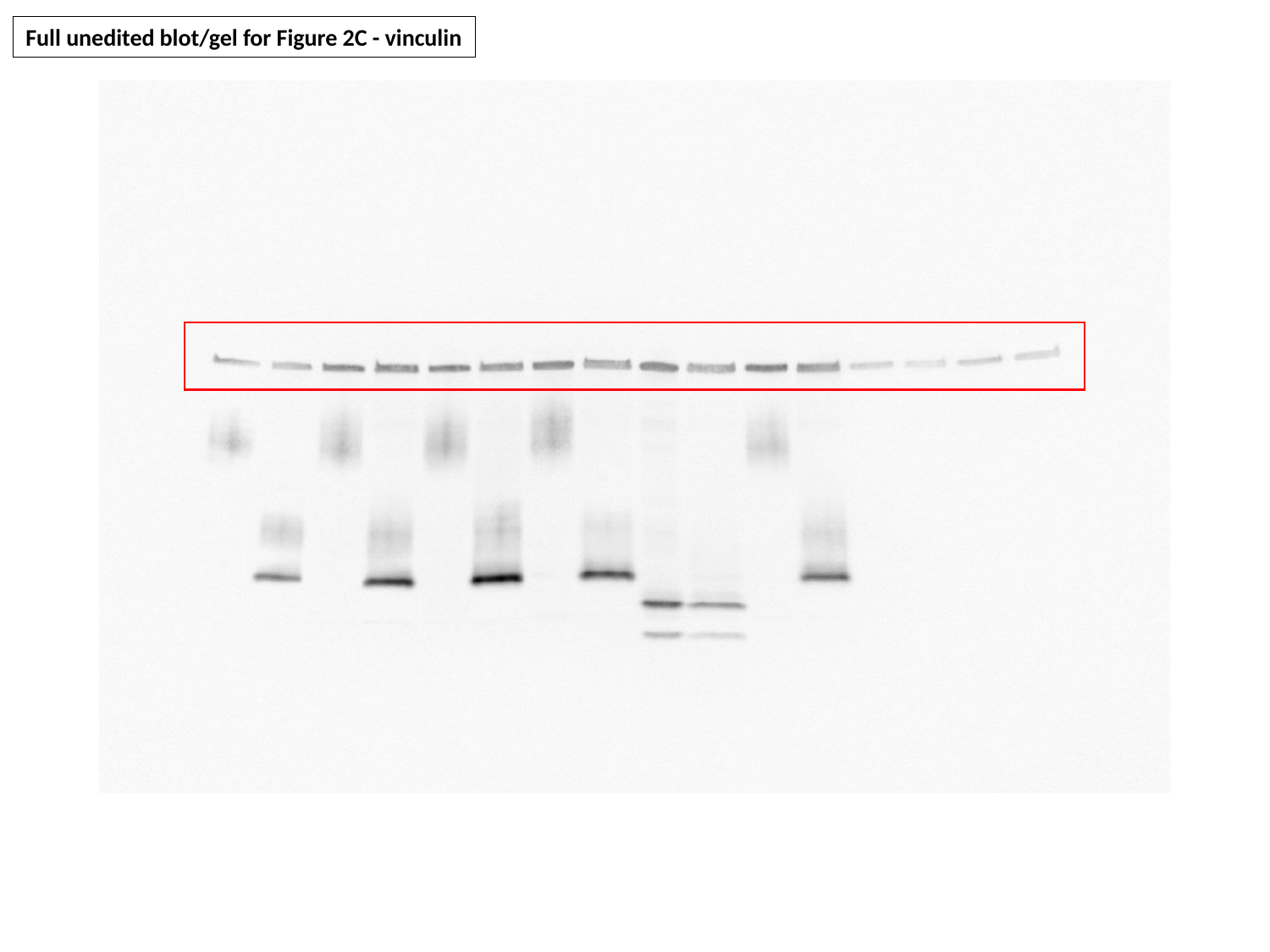

Full unedited blot/gel for Figure 2C - vinculin

## Slide 3
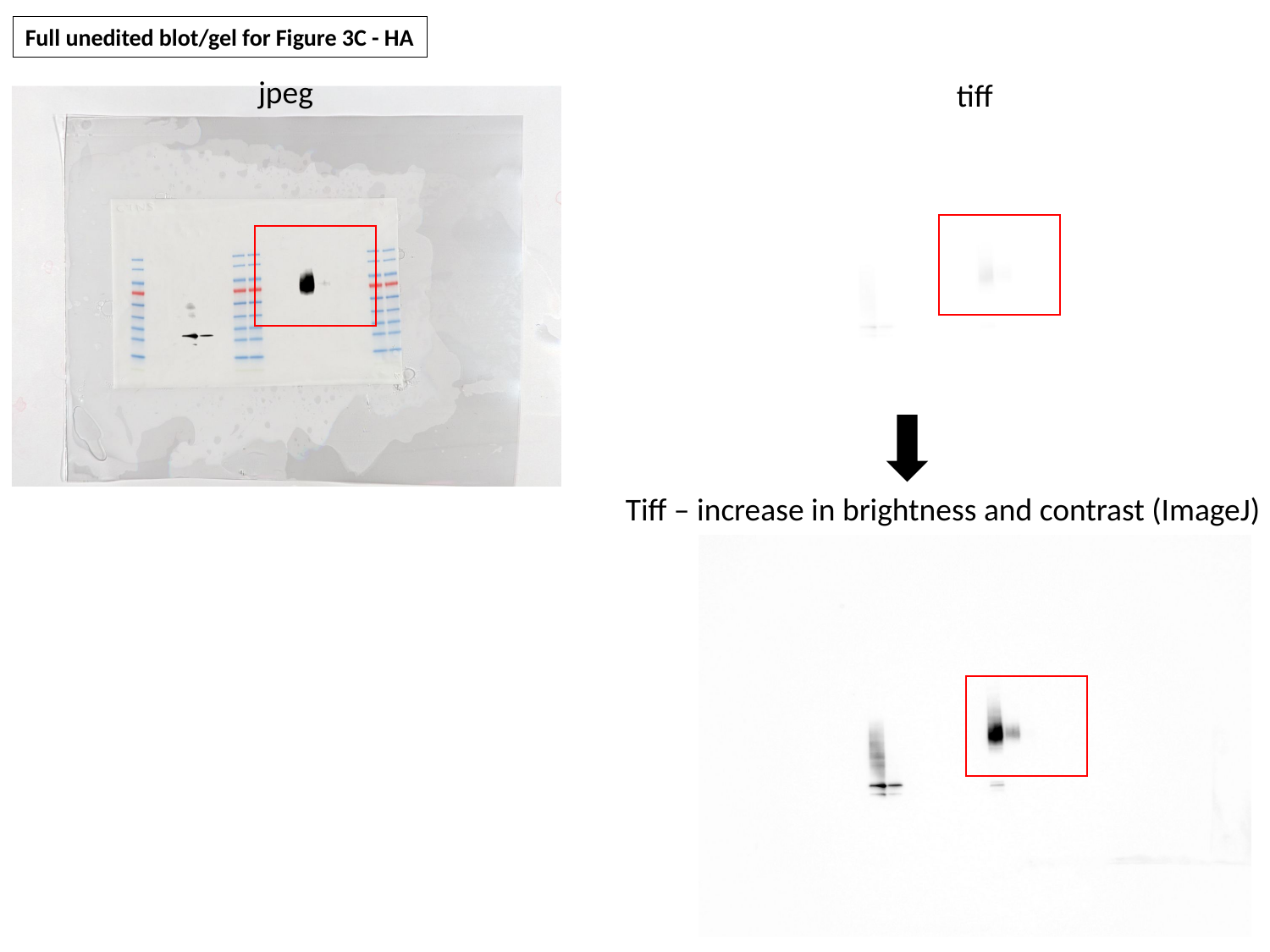

Full unedited blot/gel for Figure 3C - HA
jpeg
tiff
Tiff – increase in brightness and contrast (ImageJ)

## Slide 4
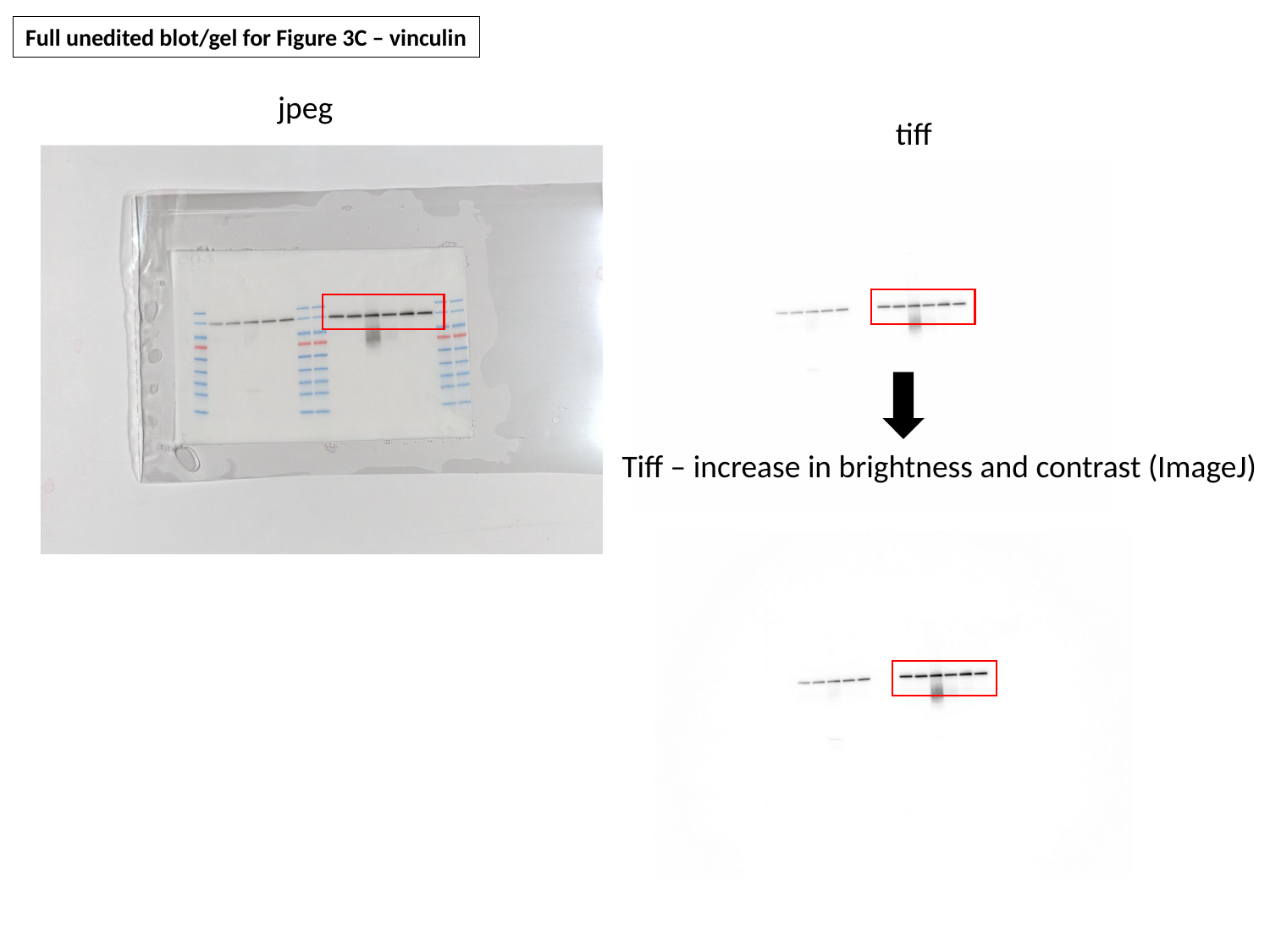

Full unedited blot/gel for Figure 3C – vinculin
jpeg
tiff
Tiff – increase in brightness and contrast (ImageJ)

## Slide 5
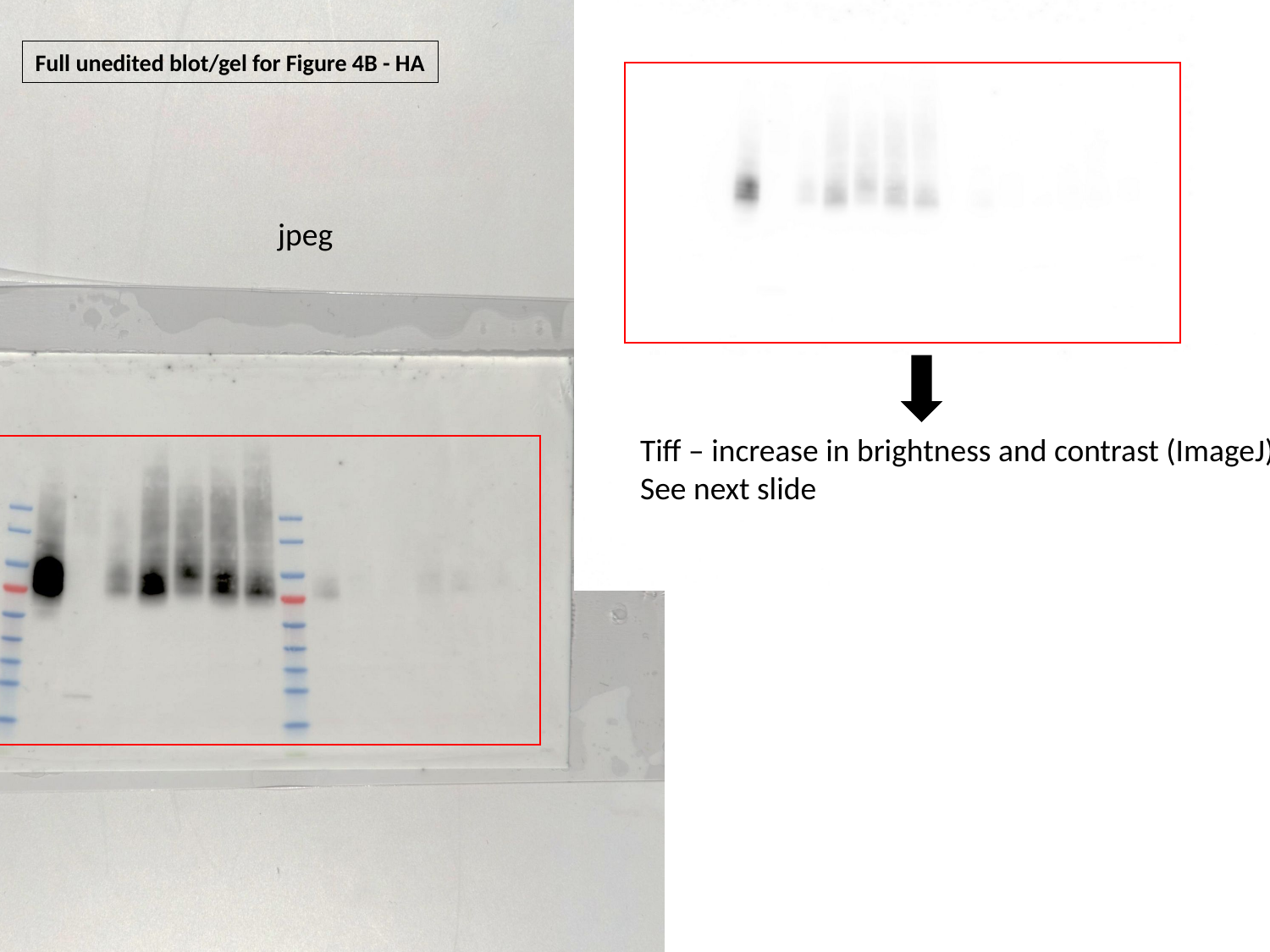

tiff
Full unedited blot/gel for Figure 4B - HA
jpeg
Tiff – increase in brightness and contrast (ImageJ)
See next slide

## Slide 6
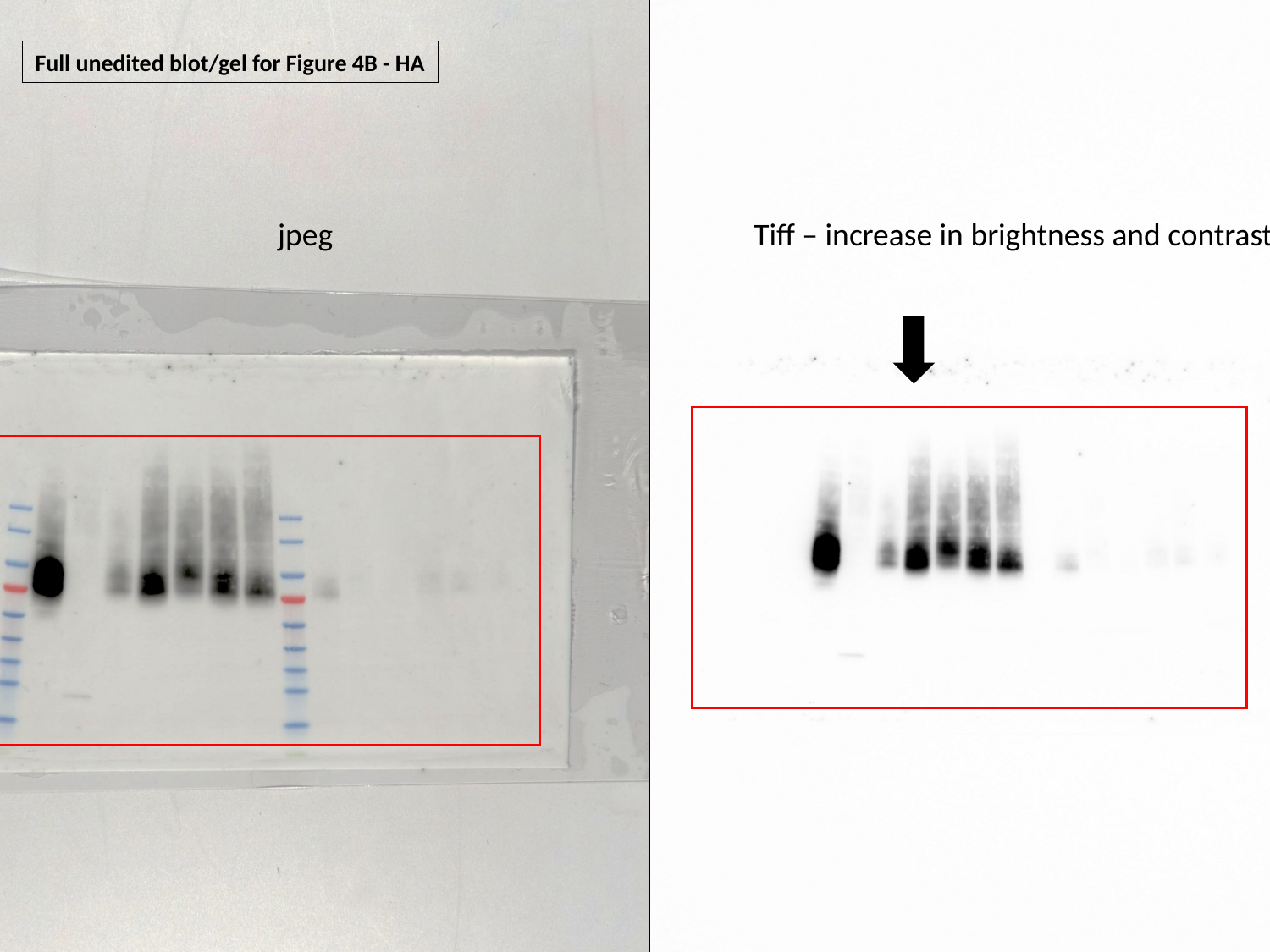

tiff
Full unedited blot/gel for Figure 4B - HA
jpeg
Tiff – increase in brightness and contrast (ImageJ)

## Slide 7
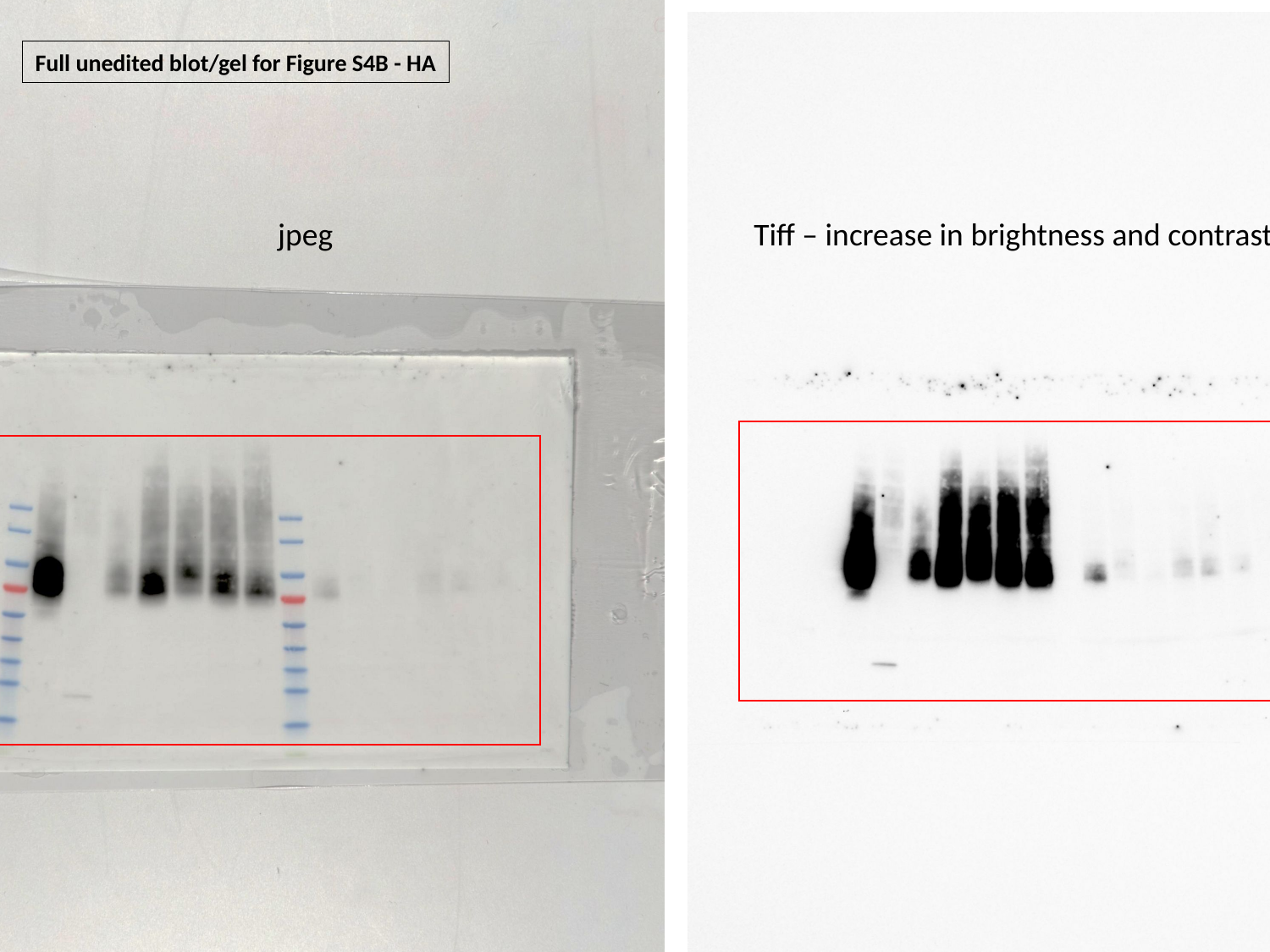

tiff
Full unedited blot/gel for Figure S4B - HA
jpeg
Tiff – increase in brightness and contrast (ImageJ)

## Slide 8
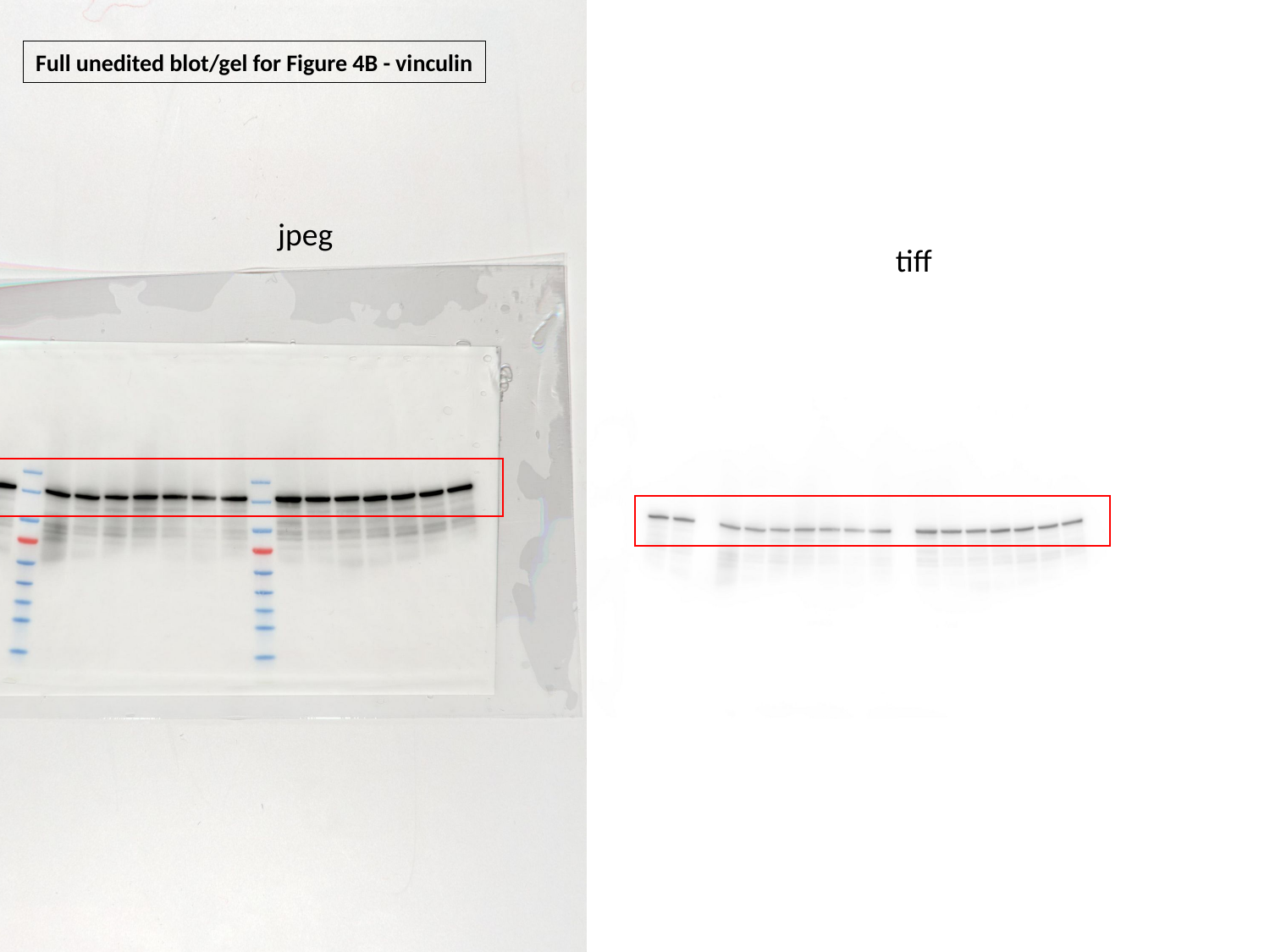

Full unedited blot/gel for Figure 4B - vinculin
jpeg
tiff

## Slide 9
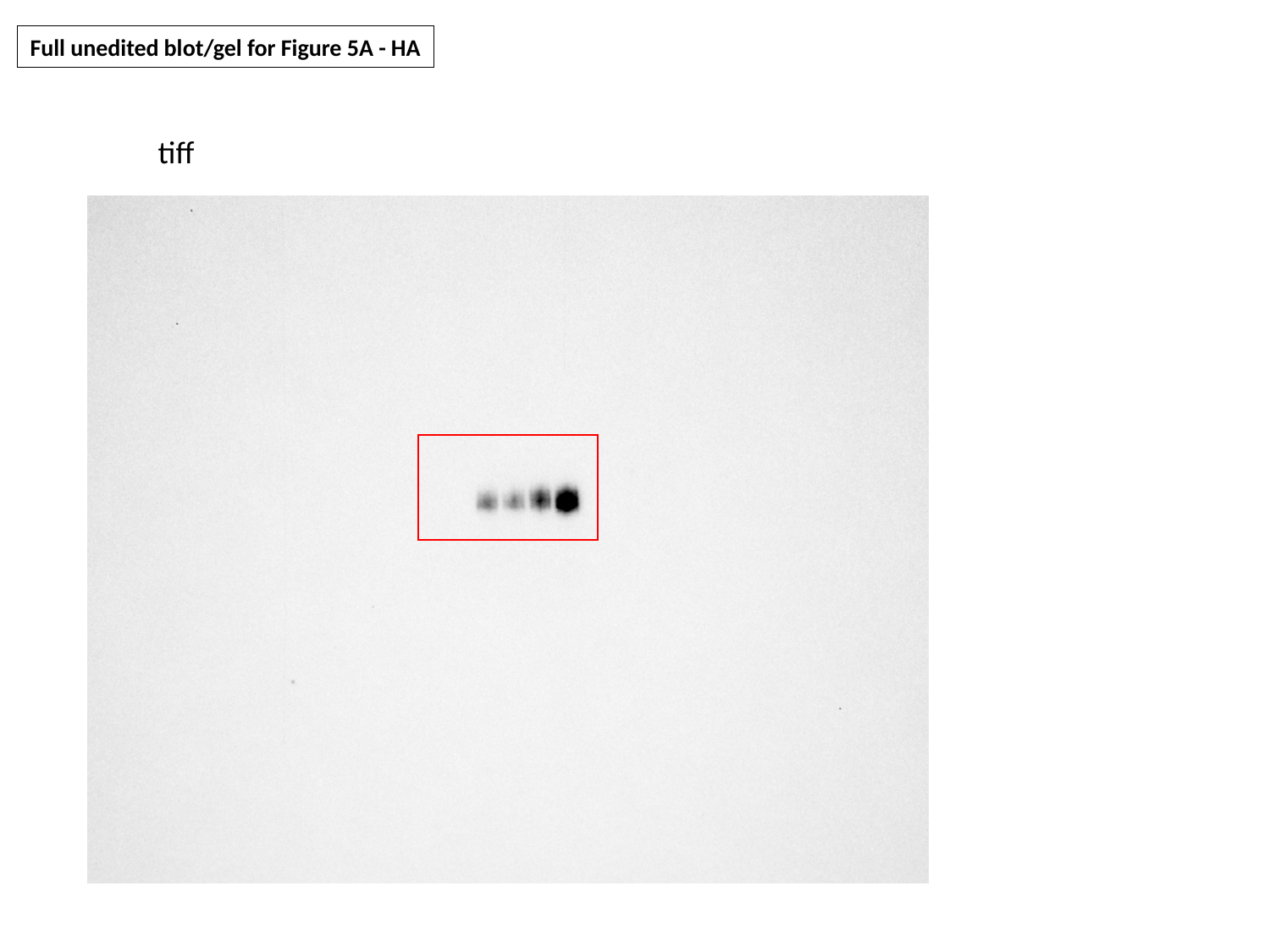

Full unedited blot/gel for Figure 5A - HA
tiff

## Slide 10
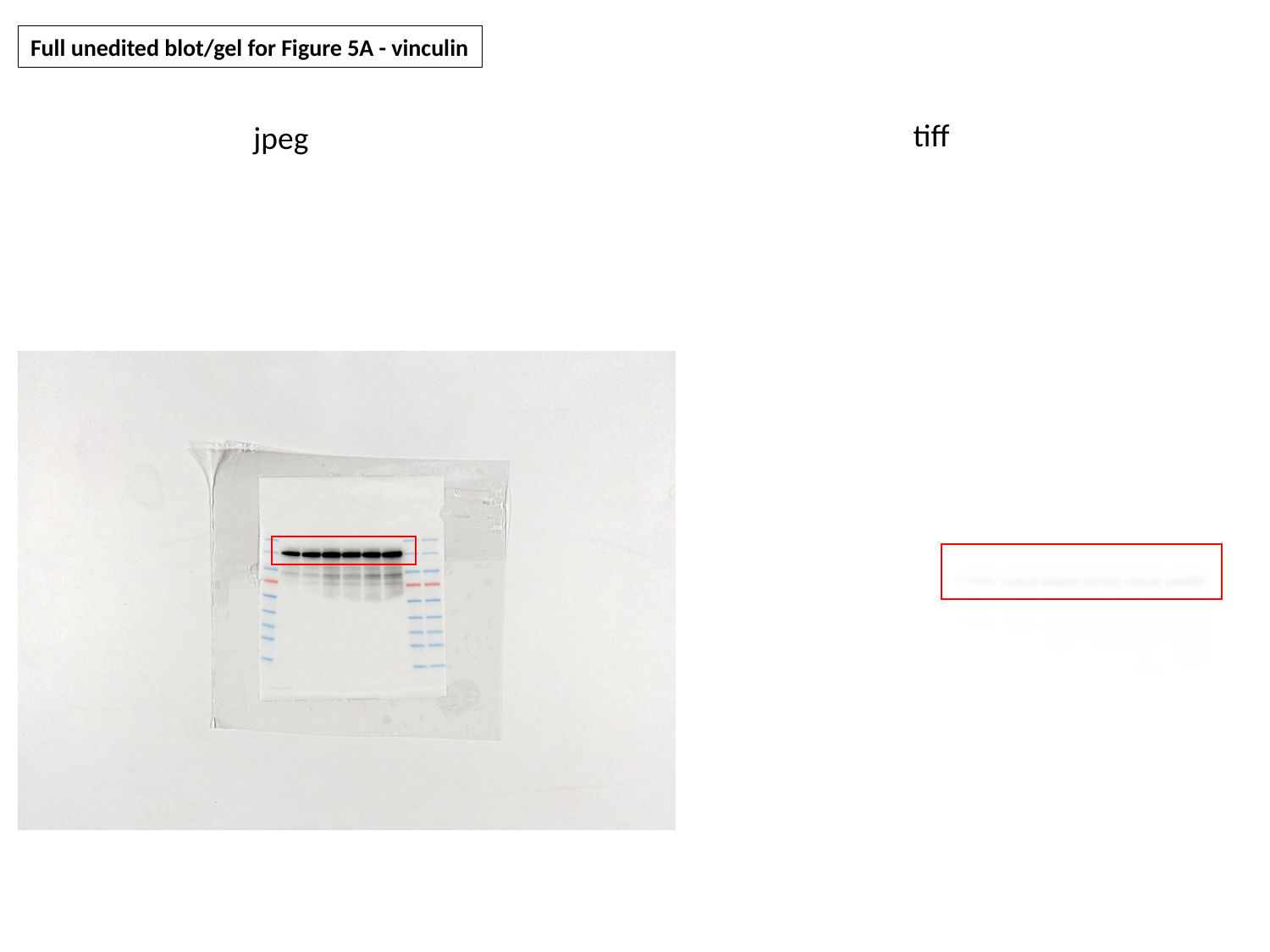

Full unedited blot/gel for Figure 5A - vinculin
tiff
jpeg

## Slide 11
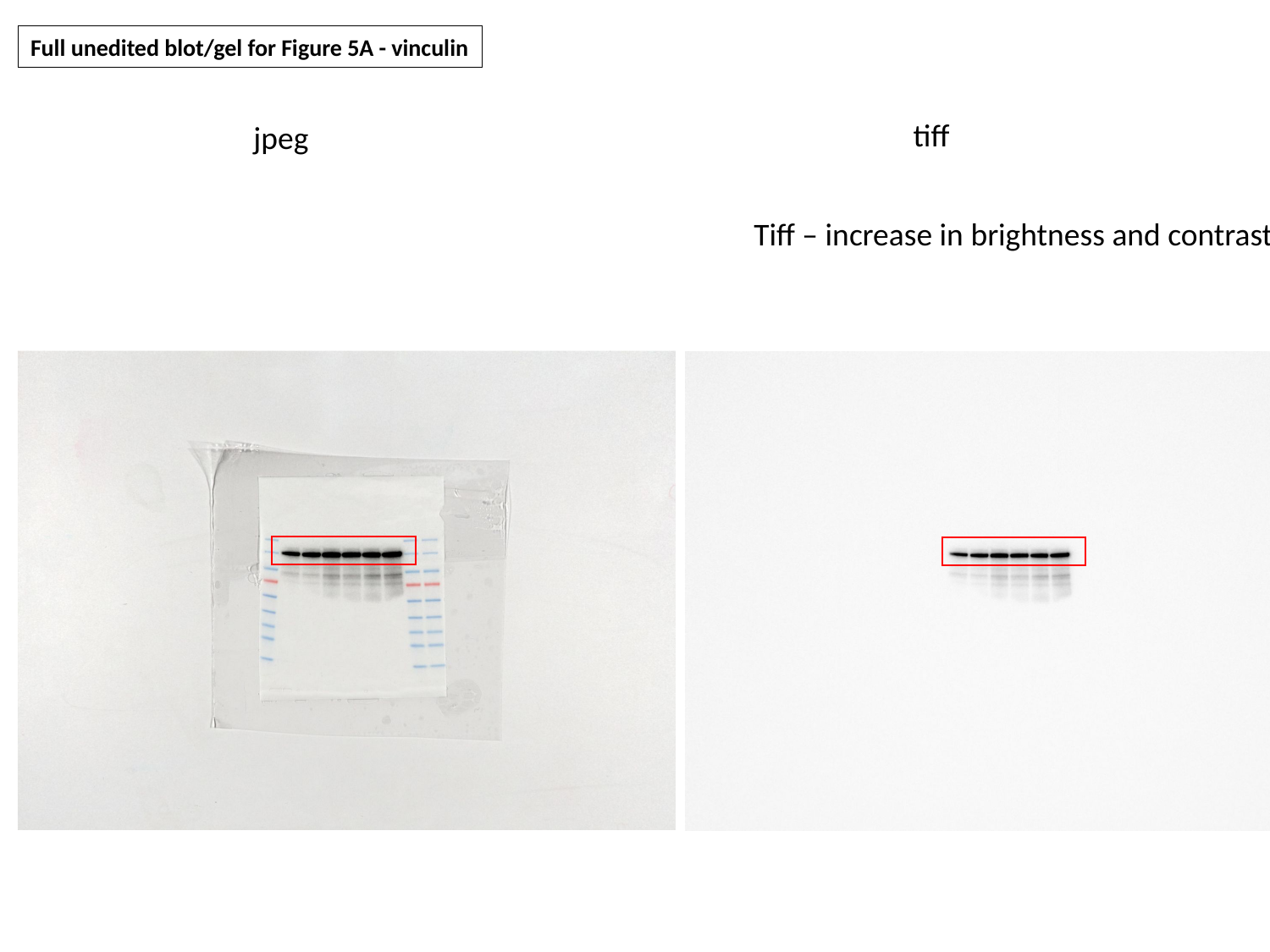

Full unedited blot/gel for Figure 5A - vinculin
tiff
jpeg
Tiff – increase in brightness and contrast (ImageJ)
