# Supplementary material for: Residual Cystine Transport Activity for Specific Infantile and Juvenile CTNS Mutations in a PTEC-Based Addback Model
Source: Cells. 2024 Apr 6;13(7):646. doi: 10.3390/cells13070646 (PMC11011962; doi:10.3390/cells13070646)
Supplement: Supplementary file 1 [file cells-13-00646-s001.zip › Supplemental_Figures-paper-CTNSmutant-V11.pdf]

Table S1. Overview of primer sequences

Target	cDNA/gDNA	Forward 5' → 3'	Reverse 5' → 3'
Woodchuck hepatitis virus WPRE	gDNA	caattccgtggtgttgtcgg	gaaggtccgctggattgagg
Hs CTNS	cDNA	ccacaggcctacatgaactt	tccactggtcgttgttgtag
Hs β-actin	gDNA	tcacccacactgtgcccatctacga	cagcggaaccgctcattgccaatgg
Hs γ-actin	cDNA	cactgagcgaggctacagctt	ttgatgtcgcgcacgattt

Table S2. Overview of primary and secondary antibodies

ANTIBODY	PROVIDER	SPECIES	DILUTION	APPLICATION
Anti-mouse Alexa 488	Invitrogen A11001	Goat	1/500	ICC
Anti-rabbit Alexa 555	Invitrogen A21429	Goat	1/500	ICC
Anti-mouse Alexa 633	Life Technologies A21050	Goat	1/500	ICC
Phalloidin Alexa 633	ThermoFisher Scientific 21840	NA	1/1000	ICC
Dapi	Life Technologies D1306	NA	1/1000	ICC
HA.11	BioLegend 901515	Mouse	1/1000	ICC
LA1	Cell Signaling 9091	Rabbit	1/200	ICC
HA.11	BioLegend 901515	Mouse	1/10 000	WB
HA (2-2.2.14)	Invitrogen 26183	Mouse	1/2000	WB
vinculin	Sigma V9131	Mouse	1/100 000	WB
anti-mouse polyclonal immunoglobulins/HRP	Agilent Dako P0260/P0447	Rabbit/goat	1/10 000	WB

FIGURE S1

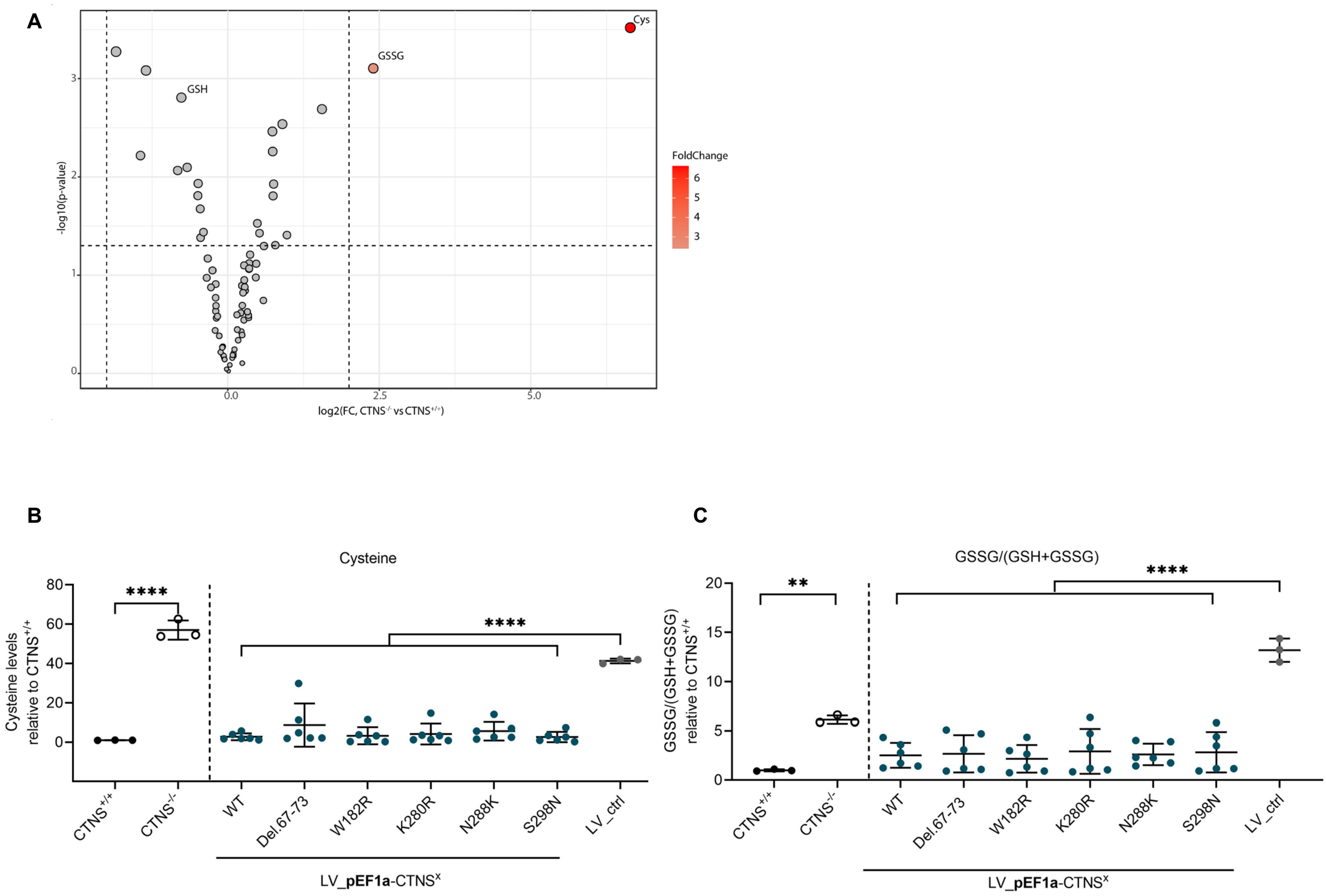

Figure S1. $CTNS^{WT}$ and $CTNS^{mutant}$ cDNA addition after LV transduction in CRISPRed $CTNS^{-/-}$ ciPTECs reduces the intracellular cysteine and redox levels.

(A) Volcano plot of metabolome changes in $CTNS^{-/-}$ ciPTECs (Metaboanalyst 6.0). Red dots show the differentially produced metabolites. Fold change threshold =4.0 and P-value threshold = 0.05; unpaired t-test.

(B) Cysteine measurement (mass spectrometry) of $CTNS^{-/-}$ ciPTECs transduced with either LV_pEF1a-$CTNS^{WT}$, LV_pEF1a-$CTNS^{mutant}$, or LV_ctrl. The data were normalized to protein content and are presented as the mean ± SD (n=3 or 6 independent metabolite extracts). Statistical testing was performed with a one-way Anova, Sidak's multiple comparison test.

(C) Redox measurement, represented as GSSG/(GSH+GSSG) (mass spectrometry) of $CTNS^{-/-}$ ciPTECs transduced with either LV_pEF1a-$CTNS^{WT}$, LV_pEF1a-$CTNS^{mutant}$, or LV_ctrl. The data were normalized to protein content and are presented as the mean ± SD (n=3 or 6 independent metabolite extracts). Statistical testing was performed with a one-way Anova, Sidak's multiple comparison test.

LV, lentiviral vector; p, promoter; WT, wild-type; LV_ctrl, LV_pEF1a-eGFP; NT, non-transduced; Cys, cysteine; GSH, reduced glutathione; GSSG, oxidized glutathione; FC, foldchange; **, p<0.01; ****, p<0.0001.

FIGURE S2

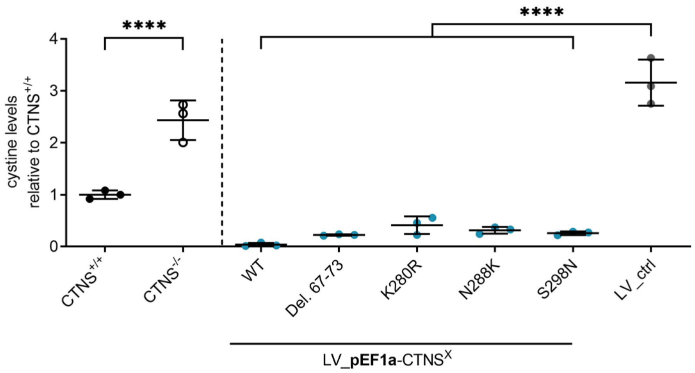

Figure S2. *CTNS^{WT} and CTNS^{mutant}* cDNA addition after LV transduction in cystinosis patient-derived fibroblasts greatly reduces the intracellular cystine levels. Cystine measurement (mass spectrometry) of CTNS$^{-/-}$ fibroblasts transduced with either LV_pEF1a-CTNS^{WT}, LV_pEF1a-CTNS^{mutant}, or LV_ctrl. The data are presented as the mean ± SD (n=3 independent metabolite extracts). Cystine (µM) was normalized to protein content (µg/µl). Statistical testing was performed with a one-way Anova, Sidak's multiple comparison test. LV, lentiviral vector; p, promoter; WT, wild-type; LV_ctrl, LV_pEF1a-eGFP; ****, p<0.0001.

Figure S3. Weaker promoters leading to lower CTNS^{WT} expression are still able to rescue the cystinosis phenotype

(A) Effect of promoter differences on eGFP expression (flow cytometry) in CTNS-/- ciPTECs transduced with lentiviral vectors LV_pEF1a-eGFP, LV_pEFS-eGFP, LV_pCTNS-eGFP . Promoter activities as MFI for ~30 %eGFP positive cells (considered as one integrated copy).

(B) Quantification of integrated copies in CTNS$^{-/-}$ ciPTECs transduced with lentiviral vectors LV_pEF1a-CTNS^{WT}, LV_pEFS-CTNS^{WT}, LV_pCTNS-CTNS^{WT} or LV_ctrl. Integrated copies were measured by quantification of the WPRE element present in the transgene construct. The data is normalized for total levels of *ACTB* and are presented as the mean ± SD (n=1 triplicates).

(C) Quantification of CTNS^{WT}-3HA protein expression in CTNS$^{-/-}$ ciPTECs transduced with lentiviral vectors LV_pEF1a-CTNS^{WT}, LV_pEFS-CTNS^{WT}, LV_pCTNS-CTNS^{WT} or LV_ctrl (n=2-3). Samples normalized for total proteins of vinculin.

(D) Confocal microscopy images of the immunofluorescence signal of CTNS^{WT}-3HA and LAMP1 in CTNS$^{-/-}$ ciPTECs transduced with either LV_pEF1a-CTNS^{WT}, LV_pEFS-CTNS^{WT}, LV_pCTNS-CTNS^{WT} or LV_ctrl. Nuclei were stained with DAPI. Scale bars are 10 µM.

(E) Cysteine measurement (mass spectrometry) of CTNS$^{-/-}$ ciPTECs transduced with LV_pEF1a-CTNS^{WT}, LV_pEFS-CTNS^{WT}, LV_pCTNS-CTNS^{WT} or LV_ctrl. The data are presented as the mean ± SD (n=1, 3 or 6 independent metabolite extracts). Cysteine (Abundance) was normalized to protein content (µg/µl).

(F) Redox measurement, represented as GSSG/(GSH+GSSG) (mass spectrometry) of CTNS$^{-/-}$ ciPTECs transduced with LV_pEF1a-CTNS^{WT}, LV_pEFS-CTNS^{WT}, LV_pCTNS-CTNS^{WT} or LV_ctrl. The data are presented as the mean ± SD (n=1, 3 or 6 independent metabolite extracts). GSSG/(GSH+GSSG) (Abundance) was normalized to protein content (µg/µl).

LV, lentiviral vector; p, promoter; MFI, median fluorescent intensity; WT, wild-type; LV_ctrl, LV_pCMV-dATP13A2; NT, non-transduced

FIGURE S3

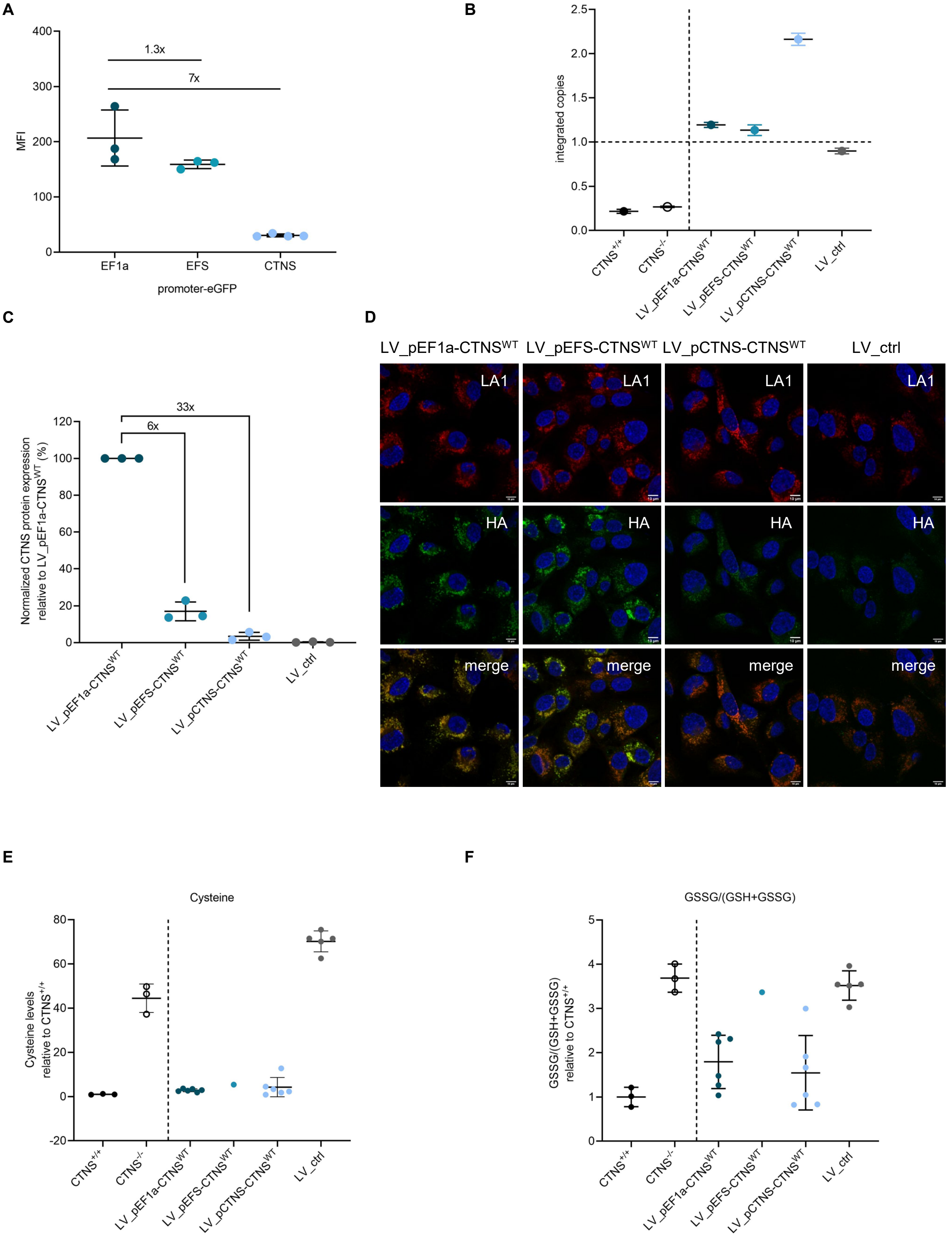
A
MFI
1.3x
7x
EF1a
EFS
CTNS
promoter-eGFP
B
integrated copies
CTNS+/+
CTNS-/-
LV_pEF1a-CTNSWT
LV_pEFS-CTNSWT
LV_pCTNS-CTNSWT
LV_ctrl
C
Normalized CTNS protein expression relative to LV_pEF1a-CTNSWT (%)
33x
6x
D
LV_pEF1a-CTNSWT
LV_pEFS-CTNSWT
LV_pCTNS-CTNSWT
LV_ctrl
LA1
HA
merge
E
Cysteine
Cysteine levels relative to CTNS+/+
F
GSSG/(GSH+GSSG)
GSSG/(GSH+GSSG) relative to CTNS+/+

FIGURE S4

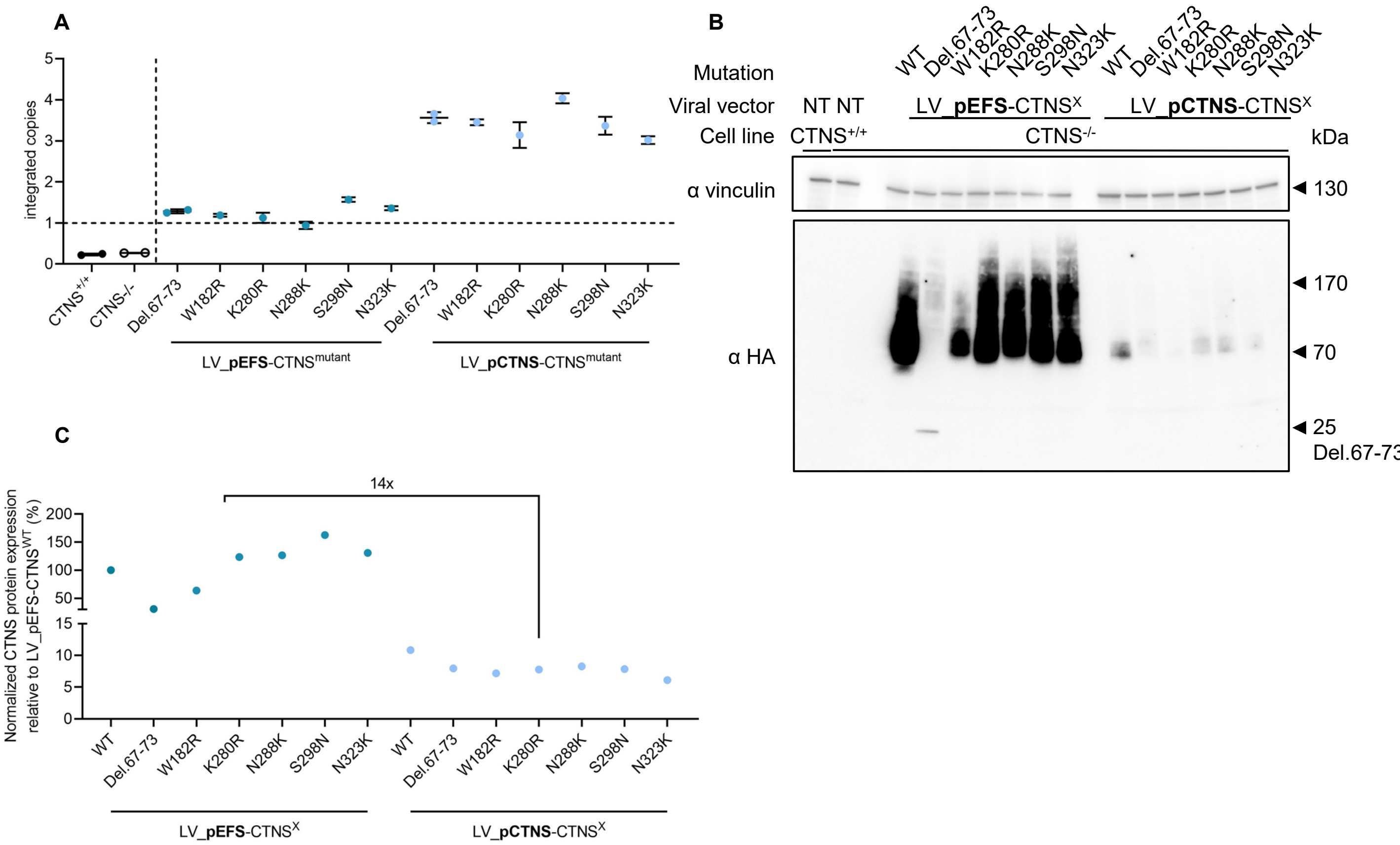

Figure S4. Different promoters showing rescue of mutants even with low activity

(A) Quantification of integrated copies in CTNS$^{-/-}$ ciPTECs transduced with lentiviral vectors LV_pEFS-CTNS^{mutant} LV_pCTNS-CTNS^{mutant} or LV_ctrl. Integrated copies were measured by quantification of the WPRE element present in the transgene construct. The data is normalized for total levels of *ACTB* and are presented as the mean ± SD (n=1 triplicates).

(B) Enhanced contrast of Figure 4B using ImageJ. Western blot analysis of CTNS$^{WT\ or\ mutant}$-3HA protein expression in CTNS$^{-/-}$ ciPTECs transduced with lentiviral vectors vectors LV_pEFS-CTNS$^{WT\ or\ mutant}$, LV_pCTNS-CTNS$^{WT\ or\ mutant}$ or LV_ctrl. Samples normalized for total proteins of vinculin.

(C) Quantification of CTNS^{mutants}-3HA protein expression via Western blot analysis of CTNS$^{WT\ or\ mutant}$-3HA protein expression in CTNS$^{-/-}$ ciPTECs transduced with lentiviral vectors vectors LV_pEFS-CTNS$^{WT\ or\ mutant}$, LV_pCTNS-CTNS$^{WT\ or\ mutant}$. or LV_ctrl (n=2). Samples normalized for total proteins of vinculin.

LV, lentiviral vector; p, promoter; WT, wild-type; LV_ctrl, LV_pCMV-dATP13A2; NT, non-transduced.

FIGURE S5

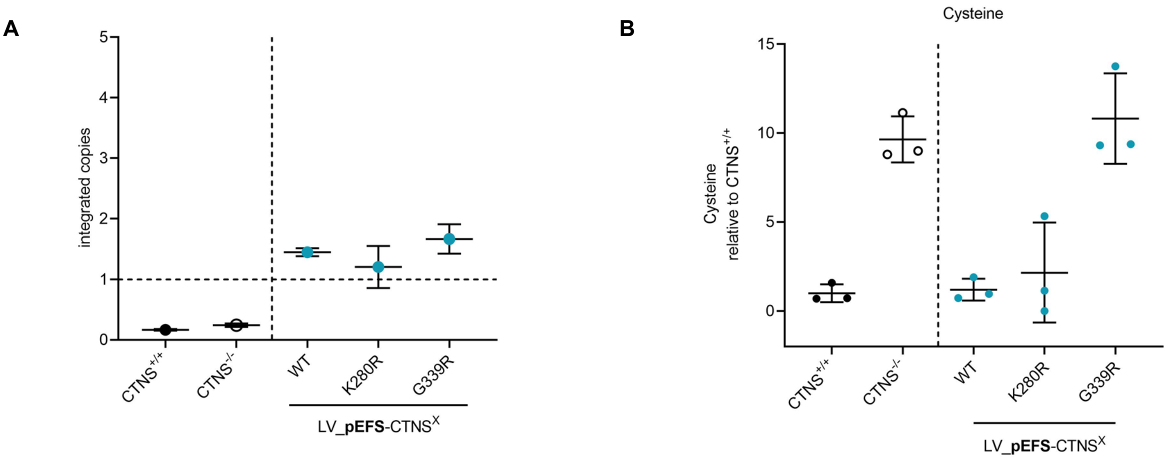

Figure 5. G339R mutant overexpression shows no rescue in cystine accumulation upon cDNA addition in CTNS$^{-/-}$ ciPTECs

(A) Quantification of integrated copies in CTNS$^{-/-}$ ciPTECs transduced with lentiviral vectors LV_pEFS-CTNS^{WT} or LV_pEFS-CTNS$^{K280R\ or\ G339R}$. Integrated copies were measured by quantification of the WPRE element present in the transgene construct. The data is normalized for total levels of *ACTB* and are presented as the mean ± SD (n=1 triplicates).

(B) Cysteine measurement (mass spectrometry) of CTNS$^{-/-}$ ciPTECs transduced with LV_pEFS-CTNS^{WT} or LV_pEFS-CTNS$^{K280R\ or\ G339R}$. The data are presented as the mean ± SD (n=3 independent metabolite extracts). Cysteine (Abundance) was normalized to protein content (µg/µl).

LV, lentiviral vector; p, promoter; WT, wild-type; ***, p<0.001; ns, nonsignificant
